# Accuracy issues involved in modeling *in vivo* protein structures using PM7

**DOI:** 10.1002/prot.24826

**Published:** 2015-06-06

**Authors:** Benjamin P. Martin, Christopher J. Brandon, James J. P. Stewart, Sonja B. Braun‐Sand

**Affiliations:** ^1^ Department of Chemistry and BiochemistryUniversity of ColoradoBoulderColorado80918; ^2^ Stewart Computational Chemistry15210 Paddington CircleColorado SpringsCO80921

**Keywords:** protein, PDB, X‐ray structures, PM7, semiempirical, errors, geometry optimization

## Abstract

Using the semiempirical method PM7, an attempt has been made to quantify the error in prediction of the *in vivo* structure of proteins relative to X‐ray structures. Three important contributory factors are the experimental limitations of X‐ray structures, the difference between the crystal and solution environments, and the errors due to PM7. The geometries of 19 proteins from the Protein Data Bank that had small *R* values, that is, high accuracy structures, were optimized and the resulting drop in heat of formation was calculated. Analysis of the changes showed that about 10% of this decrease in heat of formation was caused by faults in PM7, the balance being attributable to the X‐ray structure and the difference between the crystal and solution environments. A previously unknown fault in PM7 was revealed during tests to validate the geometries generated using PM7. Clashscores generated by the Molprobity molecular mechanics structure validation program showed that PM7 was predicting unrealistically close contacts between nonbonding atoms in regions where the local geometry is dominated by very weak noncovalent interactions. The origin of this fault was traced to an underestimation of the core‐core repulsion between atoms at distances smaller than the equilibrium distance. Proteins 2015; 83:1427–1435. © 2015 The Authors. Proteins: Structure, Function, and Bioinformatics Published by Wiley Periodicals, Inc.

## INTRODUCTION

Increasingly, properties of large biomolecules, including minimum energy structures, non‐covalent interactions of the type found in ligand binding, and reaction mechanisms, have been modeled using computational chemistry methods. For such modeling to be useful two prerequisites must be satisfied: First, the model should be as complete as possible, and second, the computational method should be sufficiently accurate that a measure of trust can be placed in its predictions. Unless these criteria are satisfied, little confidence can be placed in the results obtained from modeling simulations.

As a minimum, a realistic model would need to be chemically sensible, that is, to be as near to the conditions that are believed to exist *in vivo* as possible. This implies that the model would need to represent the solvated species, and, ideally, to represent conditions at the temperature at which biochemical processes occur. In addition, the computational method should be sufficiently versatile and efficient that common chemical and biochemical processes, such as minimum energy geometries, noncovalent interactions, and chemical reactions, could be simulated in a routine manner. These criteria are satisfied by the semiempirical method PM7^1^ as implemented in MOPAC2012.^2^ Semiempirical methods involve a quantum mechanical model of a chemical system. These methods differ from the more rigorous *ab initio* methods in that most of the computationally intensive, that is, time‐consuming, parts of Hartree‐Fock theory have been replaced by approximations that have adjustable parameters; these parameters are then adjusted so that the resulting method gives an optimized root mean square fit to a set of reference data. By using reference data derived from experimental and high level theoretical methods, a useful accuracy can be achieved, and by using approximate methods, large systems can be modeled in a practical time. For example, an unrestricted geometry optimization of a system of several thousand atoms comprising a protein, substrate, entrained small molecules, and solvent might take several hours to days on a conventional desktop computer. Such large systems cannot currently be modeled by more sophisticated methods, instead hybrid methods are used in which interesting parts of a system are modeled *ab initio* while the rest of the system is modeled using molecular mechanics methods.

PM7 has been extensively validated by comparison^1^ of reference data and PM7 results for properties such as heats of formation (Δ*H*_f_) and geometries of small molecules and organic and inorganic solids, and has also been validated for derived properties such as the energies of intermolecular interactions. In the validation, the relationship of the geometries predicted by PM7 and the geometries obtained from X‐ray analysis was investigated. This showed that although the semiempirical method reproduced interatomic distances with good accuracy, there were significant differences, typically an RMS difference of ∼1.0 Å, in the prediction of the tertiary structure. What was not investigated was the origin of these differences. Obvious candidates are as follows: (a) Inaccuracies in the semiempirical method; (b) Inaccuracies in the X‐ray analysis; (c) The experimentally determined crystal structure is not an accurate representation of the solvated species; (d) The flexibility of proteins might allow large changes in geometry to result from relatively small changes in energy.

The objective of this investigation therefore was to determine the accuracy of PM7 when used for modeling protein geometries. A problem in modeling protein chemistry is that the reference system cannot be easily defined, because, except for the smallest proteins, there are currently no methods for determining the time‐average structure of proteins *in vivo*. X‐ray analysis, even if all experimental errors were eliminated, would only provide the structure of a protein in the low‐temperature solid state. Because of the lack of a good reference geometry, for the purposes of this work it is useful to postulate a hypothetical ideal model (HIM), which can be defined as the biochemical system as it would exist *in vivo*. Under these conditions the model would be very dynamic because of thermal effects at *in vivo* temperatures, however, current quantum chemistry methods, even at the semiempirical level, are too computationally demanding to allow the use of dynamics for predicting the structure and properties of proteins. An alternative, used here, would be to model systems without including any internal energy, that is, as if they were at zero Kelvin. This would give the time‐independent potential energy surface (PES), and minima such as the starting complex and intermediates, and transition states, would then be represented by stationary points on this PES. Although ignoring thermal effects might appear to be a severe approximation, semiempirical quantum chemistry methods are parameterized to reproduce chemical properties at 298 K. As a result of this parameterization, calculated heats of formation, and therefore the PES, automatically include internal energy. For the purpose of this work, the HIM would thus be redefined as the time‐average or time‐independent structure at 298 K.

The aqueous medium presents a problem in that the lowest energy configuration is obviously that of ice, a configuration in which every water molecule is held firmly in place by hydrogen bonds. If this structure, or even if an amorphous, that is, glassy, structure, were used in simulations then the interactions between a protein molecule and the solvent molecules would be dependent on the orientation of the solvent molecules, both in terms of energy and optimized geometry. Obviously, at *in vivo* temperatures no such dependency would exist because individual water molecules would be making and breaking hydrogen bonds very rapidly, and the time‐average result would be a structureless liquid medium. This effect can be reproduced by using an implicit solvation technique in which the bulk solvent is represented as a structureless but polarizable medium through the application of a dielectric to the solvent accessible surface of a given molecule. In this work, the COSMO^3^ technique was used for representing the aqueous medium. The use of COSMO has the added advantage that the computational effort required is far less than if explicit water molecules were used.

Given that quantitative information about the HIM does not exist, the accuracy of prediction of the HIM is not possible. An alternative, which is possible, would be to determine the relative accuracies of the best experimental reference geometric data and the geometries predicted using PM7. Fortunately, the Quantum Chemical Benchmark Energy and Geometry Database^4^ (www.begdb.com) has prepared several datasets, including the S22^5^ and S66,^6^ that contain accurate equilibrium geometries and interaction energies for small molecular complexes comprised of carbon, hydrogen, nitrogen, and oxygen, and that display interactions characteristic of those found in proteins. The geometries and interaction energies were determined using high level electronic structure methods such as an augmented coupled cluster method with a complete basis set (CCSD(T)/CBS)^7^ and second‐order Møller‐Plesset perturbation theory (MP2).^8^ These methods build upon Hartree‐Fock theory to attempt to describe electron correlation. Use of these expensive (that is, time‐consuming) methods was necessary to accurately capture the weak dispersion interactions occurring between the dimers studied in the S22 and S66 datasets.

The Protein Data Bank^9^ provides a wealth of information on the structure of proteins. For simulation work the structures in the PDB have two limitations. First, because of experimental constraints, the structures generated are only a good approximation to the hypothetical true crystal structure, and second, even if the true crystal structure were available it would only be an approximation, albeit a good one,^10^ to the geometry adopted by the protein in solution. The first of these limitations—errors arising from experimental constraints—can be minimized by selecting from the PDB only proteins that have high accuracy; such protein structures could then be assumed to be the best experimental approximation to the structure found in solution.

Determining a measure of geometry's accuracy is by no means obvious. The simplest measure of accuracy, the differences between the HIM geometry and the geometries predicted by PM7 or the geometries from the PDB, is not suitable for several reasons, of which the most important is the lack of HIM reference geometry. In addition, and possibly more important, even if the idealized geometry were available, comparison of the HIM to X‐ray structure would not agree all that favorably because geometric excursions necessarily occur^10^ in going from the crystal to solution phase as crystal packing forces are relieved. Compared with protein primary and secondary structures, protein tertiary structure is often very flexible, and as environmental conditions change, for example in going from solution phase to crystal phase, it is possible for large geometric changes to occur, with these changes being accompanied by only small changes in energy. Simply comparing HIM and X‐ray geometries would therefore reflect badly on X‐ray structures, even if the X‐ray geometry was exact, that is, if there was no error attributable to experimental procedure.

An alternative, and more useful, measure of accuracy can be constructed from changes in Δ*H*_f_. This approach makes use of the fact that force constants for atom pairs that are covalently bound are much larger than those involved in nonbonded interactions, and thus contribute more to the Δ*H*_f_. Starting with the HIM structure and a hypothetical completely accurate computational method, that is, a method that could exactly reproduce the HIM structure, if a small geometric error were to be introduced in a covalent bond length, then the increase in Δ*H*_f_ due to the relative motion of the two atoms would be much greater than if a geometric error of the same magnitude were to occur between two well‐separated atoms. Differences between a geometry generated by a theoretical method and the HIM could then be expressed in terms of energy, with the contribution to the energy increase being considerably larger for errors in relative positions of strongly interacting than for weakly interacting pairs of atoms. Because the Δ*H*_f_, a scalar quantity, includes contributions from all pairs of atoms, it provides a simple measure of distortion that automatically accounts for important distortions such as changes in bond lengths and de‐emphasizes less important distortions such as changes in non‐bonded and chain‐chain distances. Changes in Δ*H*_f_ thus provide a more meaningful measure of distortion than simple geometric changes.

Unfortunately, because the HIM geometry is not available for study, calculation of the change in Δ*H*_f_ between a predicted or experimentally determined geometry and the HIM geometry is not possible. However, it can be shown that if the error in one or other of these quantities can be obtained, an estimate can be made of the other. It will also be shown that a systematic study of errors in small molecules and noncovalently bound small molecule pairs can be used to obtain a value that reflects the average per‐atom error in PM7. Using this quantity, an estimate can then be made of the difference between the HIM and X‐ray geometries. Given both estimates, expressed in units of energy, the ratio of the two—a dimensionless quantity—can then be obtained. This ratio then represents the relative accuracies of the predicted and experimental geometries. The objective of this work is not to quantify individual errors in calculated or experimental geometries; rather it is to compare the accuracy with which PM7 can predict the *in vivo* environment of proteins relative to X‐ray geometries of proteins. Finally, it should be reiterated that because X‐ray analyses give rise to crystal structures, not solution‐phase structures, the term “accuracy” should not be construed as suggesting any error in X‐ray structures. Obviously, even if all experimental error were eliminated, an X‐ray structure of a protein would only be a good approximation to the geometry in the solution‐phase.

## MATERIALS AND METHODS

Entries in the PDB can be regarded as experimentally determined structures. Each entry has an assigned quality, and for the purpose of comparison of experimental and theoretical accuracy, only high resolution (≤1.0 Å) structures were used. In all, 19 proteins that had been deposited recently in the PDB were selected, ranging in size from 21 to 279 residues. All nonprotein moieties present in the original PDB file, such as water and small organic compounds, and so forth, were retained. Most of the entries did not include hydrogen atoms, so all entries were hydrogenated using the ADD‐H utility in MOPAC2012, with those entries that already included hydrogen atoms first being dehydrogenated. In order to avoid issues relating to ionization, all ionizable sites were, by convention, protonated according to their respective p*K*_a_ in water. When hydrogen atoms were added, their positions were initially defined by the atom they were attached to, and their orientation was defined based on the hybridization of the atom. Many proteins contained entrained water molecules, each of which was represented by a single oxygen atom. Because other atoms were not present to help define the positions of the hydrogen atoms attached to the oxygen, when the hydrogenation process was performed all such water molecules had exactly the same orientation. This obviously gave rise to an unrealistic structure. To rectify this, the positions of the added hydrogen atoms were then optimized while the positions of all other atoms were held constant. During this optimization process a small number of salt bridges spontaneously formed in some proteins. Two single‐point calculations were then run on each protein, one with and one without implicit solvation and with the dielectric constant set to that of water, that is, ε**_r_**(H_2_O) = 78.4. The resulting structures and heats of formation were then used in the subsequent work.

Next, the all‐atom geometries of each of the 19 proteins were then optimized using PM7 both in the gas phase and with implicit solvation with the dielectric constant again set to that of water, that is, *ε* **_r_**(H_2_O) = 78.4. No geometric or other constraints were used.

## RESULTS

For each protein, these calculations gave rise to four different Δ*H*_f_: The unoptimized PDB heavy‐atom structure both with and without solvation, Δ*H*_f_(PDB) and Δ*H*_f_(PDB_Aq_), and the fully optimized structure, again with and without solvation, Δ*H*_f_(PM7) and Δ*H*_f_(PM7 _Aq_), as shown in Table 1. The difference between Δ*H*_f_(PDB) and Δ*H*_f_(PM7) would then represent the total geometric change of the protein in going from the reported crystal structure to the predicted PM7 structure *in vacuo*, and the difference between Δ*H*_f_(PDB_Aq_) and Δ*H*_f_(PM7_Aq_) would similarly represent the change in going from the crystal structure to the predicted PM7 structure in aqueous media. The use of total changes in geometry as a measure of accuracy would make comparison of different proteins difficult because of the wide range of sizes spanned by the proteins used, so to allow the energies of the various species to be compared all calculated heats of formation were divided by the number of atoms in the species. The geometric changes per atom, *ε*(tot_g_), and *ε*(tot_Aq_), are defined as shown in Eqs (1) and 2:

(1) $$ɛ\left({\text{tot}}_{g}\right)\;=\;\left(\Delta H_{f}\left({\text{PDB}}_{g}\right)-\Delta H_{f}\left({\text{PM}7}_{g}\right)\right)/n,$$

and

(2) $$ɛ\left({\text{tot}}_{\text{Aq}}\right)\;=\;\left(\Delta H_{f}\left({\text{PDB}}_{\text{aq}}\right)-\Delta H_{f}\left({\text{PM}7}_{\text{aq}}\right)\right)/n,$$

where *n* is the total number of atoms in the system. Values for the per atom changes in energy are shown in Table 2.

**Table 1:** Heats of Formation, (Δ*H*_f_) in kcal mol^−1^, of Single‐Point Gas Phase, Δ*H*_f_(PDB_g_), and Solvated, Δ*H*_f_(PDB_aq_), Protein Structures and Fully Optimized Gas Phase, Δ*H*_f_(PM7_g_), and Solvated, Δ*H*_f_(PM7_aq_), Structures

PDB ID	Residues	Atoms	Resolution (Å)	Δ*H*_f_(PDBg)	Δ*H*_f_(PDBaq)	Δ*H*_f_(PM7g)	Δ*H*_f_(PM7aq)
3NIR	48	933	0.48	−9677.2	−10043.6	−10588.31	−10838.52
3W5H	272	6142	0.78	−60719.18	−63125.27	−66451.1	−68066.68
3W7Y	51	2401	0.92	−26334.05	−27663.67	−28741.15	−29467.43
3WCQ	97	1715	0.97	−13737.64	−15306.84	−15253.8	−16812.44
3WDN	125	2818	0.86	−31166.42	−33656.06	−33933.21	−35935.5
3ZOJ	279	4754	0.88	−32530.8	−34379.14	−36033.83	−37029.43
4AQO	92	1653	0.99	−15879.93	−16890.7	−17594.74	−18379.29
4AR6	54	1235	0.92	−13883.15	−15511.66	−15190.45	−16644.27
4BCT	201	3966	0.98	−38694.79	−39895.79	−42054.08	−42859.72
4BY8	21	296	0.94	−1306.82	−1426.6	−1640.21	−1743
4EIC	93	1742	0.84	−15171.68	−16249.14	−16669.42	−17557.05
4FRC	260	4963	0.98	−41891.22	−43962.08	−46017.09	−46922
4FU5	260	5018	0.98	−40137.78	−43578.26	−44362.36	−47569.54
4G78	152	2802	0.92	−22818.52	−25191.37	−26087.12	−27525.25
4HGU	40	812	0.98	−7900.6	−8596	−9088.4	−9353.19
4HS1	85	1586	0.87	−11805.16	−13040.06	−13879.69	−14204.75
4KQP	232	4844	0.95	−50179.49	−52062.3	−54089.1	−55551.98
4LFS	35	803	0.97	−7126.4	−7851.03	−7859.9	−8328.35
4MZC	111	2223	0.95	−19,006	−20433.1	−20963.86	−21833.4

**Table 2:** Total Change in Δ*H*_f_ per Atom for Gas, *ε*(tot_g_), and Solution, *ε*(tot_aq_), Phase Resulting from Geometry Optimization in kcal mol^−1^ atom^−1^

PDB ID	*ε*(tot_g_)	*ε*(tot_aq_)
3NIR	0.98	0.85
3W5H	0.93	0.80
3W7Y	1.00	0.75
3WCQ	0.88	0.88
3WDN	0.98	0.81
3ZOJ	0.74	0.56
4AQO	1.04	0.90
4AR6	1.06	0.92
4BCT	0.85	0.75
4BY8	1.13	1.07
4EIC	0.86	0.75
4FRC	0.83	0.60
4FU5	0.84	0.80
4G78	1.17	0.83
4HGU	1.46	0.93
4HS1	1.31	0.73
4KQP	0.81	0.72
4LFS	0.91	0.59
4MZC	0.88	0.63
Average	0.98	0.78

### Partitioning geometric changes into pm7 and X‐ray components

Having calculated the total geometric change that would occur in going from the original X‐ray structure to the fully optimized structure, the next step would be to partition this change into two components: the change attributable to going from the X‐ray structure to the HIM, *ε*(PDB_g_), and the change in going from the PM7 structure to the HIM, *ε*(PM7_g_). This task can be simplified by using the fact that the two changes are completely uncorrelated; that is, errors in PM7 can have no influence on the geometric changes that would occur in going from the X‐ray structure to the HIM. Given that the two error components are orthogonal, the total change can then be expressed as a vector addition, as shown in Eq. (3):

(3) $$ɛ{\left({\text{tot}}_{g}\right)}^{2}=\;ɛ{\left({\text{PDB}}_{g}\right)}^{2}+\;ɛ{\left({\text{PM}7}_{g}\right)}^{2},$$

with equivalent terms for the solvated species, Eq. (4):

(4) $$ɛ{\left({\text{tot}}_{\text{Aq}}\right)}^{2}=\;ɛ{\left({\text{PDB}}_{\text{Aq}}\right)}^{2}+\;ɛ{\left({\text{PM}7}_{\text{Aq}}\right)}^{2}.$$

There are two components to the geometric change attributable to the X‐ray structure: errors in the geometry due to experimental causes and the change in geometry arising from removing the structure from the crystal to the gaseous or aqueous phase. In principle, these components should be regarded as uncorrelated, thus contributing to the total change as their vector sum, but, because there is no way to separate the two quantities, for convenience they are considered as a single quantity.

All that remains now is to evaluate one of the two quantities on the right hand side of these equations. If no assumptions are made, this step would be intractable, but if the assumption is made that most of the geometric error attributable to PM7 is caused by interactions involving only the nearest few neighbors of each atom, or, put another way, that energy contributions from changes in long‐range interactions are negligible, then it is possible for the error due to PM7 to be calculated.

Intermolecular interaction energies have been calculated using very accurate methods such as CCSD(T)/CBS.^4^, ^5^ Two popular reference data sets of such calculations are the S22^5^ and S66^6^ sets; in these sets both the interaction energies and the geometries of the species involved were reported. For the work reported here, these geometries can be regarded as definitive, that is, as reference geometries, and therefore any deviation from them can be regarded as a geometric error. This, then, provides a method for determining the geometric error attributable to PM7.

Of the 88 systems in S22 and S66, 73 systems that did not include a nucleic acid were used. Systems with nucleic acids were excluded because none of the 20 common residues that occur in proteins contains heterocycles of the type found in nucleic acids. A single‐point calculation using PM7 was performed on each reference geometry; this was then followed by a complete geometry optimization. Using the definition described earlier, the difference in energy of these two geometries thus represents the total geometric error due to using the PM7 method. This was normalized through division by the number of atoms in the system to yield a value of *ε*(PM7_g_) for that system. By averaging the values of *ε*(PM7_g_) for all 73 species, an estimate of 0.12 kcal mol^−1^ atom^−1^ was obtained for the mean value of *ε*(PM7_g_). Solvation effects would be unlikely to change this quantity significantly, the effects of solvation being similar for both high level and semiempirical methods, and therefore cancelling when differences were calculated, so for convenience the same value was used for solvated proteins, that is, *ε*(PM7_aq_) = 0.12 kcal mol^−1^ atom^−1^.

From a knowledge, of the error attributable to PM7 and the total change in energy in going from the PDB to the PM7 geometry, the geometric change that would occur in going from the PDB to the HIM can now be calculated using Eqs. (3) and 4. Values of these geometric changes are given in Table 3.

**Table 3:** The Change in Δ*H*_f_ per Atom for Gas, *ε*(PDB_g_), and Solution, *ε*(PDB_aq_), Phase in Moving from the PDB Geometry to the HIM in kcal mol^−1^ atom^−1^

PDB ID	*ε*(PDB_g_)	*ε*(PDB_aq_)
3NIR	0.97	0.84
3W5H	0.93	0.80
3W7Y	1.00	0.74
3WCQ	0.88	0.87
3WDN	0.97	0.80
3ZOJ	0.73	0.54
4AQO	1.03	0.89
4AR6	1.05	0.91
4BCT	0.84	0.74
4BY8	1.12	1.06
4EIC	0.85	0.74
4FRC	0.82	0.58
4FU5	0.83	0.79
4G78	1.16	0.82
4HGU	1.46	0.92
4HS1	1.30	0.72
4KQP	0.80	0.71
4LFS	0.91	0.58
4MZC	0.87	0.62
Average	0.97	0.77

The change attributable to errors in PM7 is 0.12 kcal mol^−1^ atom^−1^.

### Other errors in pm7

**Figure 1:**
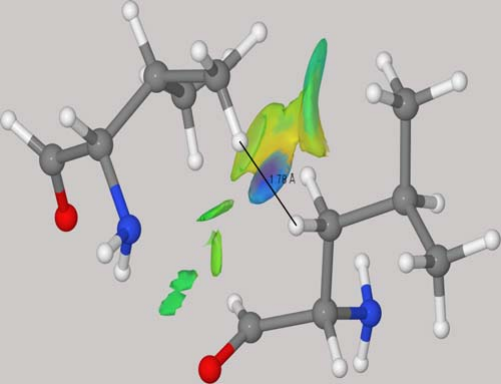
Nonbonding close contact between hydrogen atoms from Leu104 and Val110 of 4MZC that was generated during optimization with PM7. The optimized interatomic distance was 1.78 Å. The noncovalent interaction surface as generated by Jmol version 14.2.7 is shown in the figure (Jmol: an open‐source Java viewer for chemical structures in 3D. http://www.jmol.org/s).

**Figure 2:**
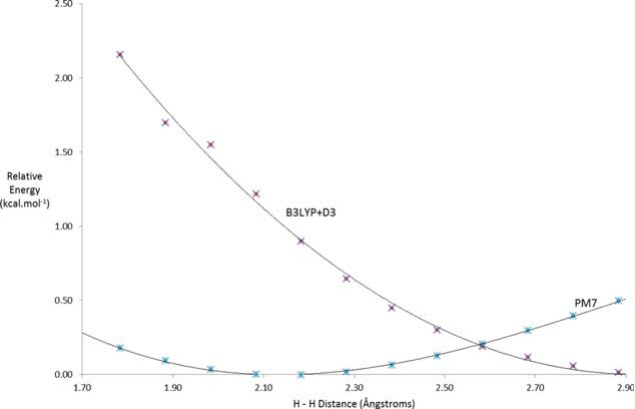
Relative heat of formation for leucine and valine at different H ‐ H separations. See text for details. [Color figure can be viewed in the online issue, which is available at wileyonlinelibrary.com.]

To investigate the energetics of this phenomenon, PES were sampled, one for the DFT functional, the DFT‐D3 method^11^ that employs Grimme's dispersion correction, with the 6‐31G basis set, and another for PM7 with the COSMO solvation option, representing the interaction energy of the two isolated amino acids at varying distances (Fig. 1). Results for the DFT functional B3LYP^12^ with the D3 correction, and for PM7 with the COSMO solvation option are shown in Figure 2. Inspection of these PES shows that the minimum for B3LYP occurs at about 2.9 Å, and that for PM7 occurs at about 2.1 Å. This difference, about 0.8 Å, was responsible for the reduced inter‐residue distance in proteins. The position of the minima is determined by two competing energies, the hard core‐core repulsion or steric effect and the softer dispersion energy, that is, the van der Waals attraction. PM7, then, either underestimates the core‐core repulsion or overestimates the vdW attraction, or both.

For further verification, and to estimate the extent to which PM7 would generate anomalous geometries, the on‐line utility MolProbity,^13^, ^14^ popular for validating experimentally determined X‐ray structures, was used. Inspection of optimized models revealed a single, consistent fault in the PM7 geometry, a fault that was not present in the original geometry, and therefore could only be caused by a defect in the PM7 method. In each of the optimized structures examined, an unrealistically short nonbonding hydrogen–hydrogen separation was found. This error only occurred between residues where there were no interactions between them other than van der Waals forces. An example of such an unrealistic interaction is provided by the residues Leu_104_ and Val_110_ in the protein 4MZC. One of these residues, Leu, was positioned near the end of a short alpha helix, the other, Val, was just outside the helix. After adding hydrogen atoms to the original structure, the shortest H–H distance between the two side‐chains was 2.2 Å. When 4MZC was optimized using PM7, this distance decreased to 1.78 Å. This is shown in Figure 1.

MolProbity can identify unfavorable steric contacts, called “clashes,” which, if present, would be indicative of a poor structure. The number of clashes in a particular system is then used in generating a “clashscore,” with a higher clashscore indicating a poorer model. Hydrogen atoms for each of the 19 proteins were removed and replaced by Molprobity at electron cloud positions and clashscores were calculated with no side‐chain flips. This scheme was adopted to preserve the heavy atom geometry and to avoid a penalty resulting from the spontaneous formation of salt bridges during optimization. Two clashscores were generated for each protein: one for the PDB structure after hydrogen atoms were added and their positions were optimized using MOPAC2012 while the rest of the geometry was frozen, and one after the entire geometry of the system was optimized.

In Table 4, a comparison of the clashscores for the completely optimized geometries with the clashscores for the same systems when only the positions of the hydrogen atoms were optimized, showed that there was a large increase in the average clashscore when moving to the fully optimized structures. This confirmed the consistency of the discovered fault but was surprising, in that it had been expected that when geometry optimization was performed, all strains would be relieved and the resulting geometry would be expected to have fewer steric faults. Instead, in several cases, the clashscores increased, often by a large amount.

**Table 4:** Clashscores for Protein Structures Predicted using PM7

Proteins	H Only	All
3NIR	0.0	20.0
3W5H	6.8	25.4
3W7Y	4.5	22.2
3WCQ	6.2	45.5
3WDN	5.2	18.6
3ZOJ	1.0	17.5
4AQO	8.1	22.6
4AR6	7.4	19.9
4BCT	4.1	20.4
4BY8	10.6	3.5
4EIC	0.0	18.4
4FRC	1.8	24.6
4FU5	2.3	36.6
4G78	13.3	17.9
4HGU	1.8	22.8
4HS1	4.0	12.7
4KQP	2.6	25.3
4LFS	0.0	10.4
4MZC	5.2	14.6
Average	4.5	21.0

H Only: After optimization of hydrogen atom positions only. All: After unconstrained optimization.

## DISCUSSION

An attempt has been made to estimate the accuracy of prediction of the geometries of protein systems *in vivo*, using the semiempirical method PM7. Every step in this process was hindered by the complexity of the issues involved. Even the definition of “accuracy” was by no means obvious. Proteins can undergo large geometric changes when even small changes are made to their environment, and the assumption could not be made that the shape of a protein in a crystal was the same as that in solution. An investigation^10^ of the distortion due to crystal packing forces concluded that “For different crystal forms, the limit of accuracy [C(alpha), root‐mean‐square deviation] is ∼0.8 Å for the entire protein, which includes ∼0.3 Å due to crystal packing.” The authors note that the crystal environment exerts a clear influence on “backbone conformations, hinge‐like motions and side‐chain conformations.” That investigation focused on the geometric change caused by the crystal environment, not on the energetics involved, nevertheless, the implication was that the energy required for the crystal packing distortion must be very small.

Two issues relevant to the *in vivo* environment are temperature effects and the aqueous medium. Semiempirical methods are parameterized to reproduce chemical properties at 298 K; as a result, internal energy terms associated with heating the system from 0 K to *in vivo* temperatures are automatically included. At *in vivo* temperatures, water exists in the liquid phase, and if explicit water were used, statistical averaging would need to be performed to eliminate the small, but not insignificant, effects of nonequilibrium, transient, solvent molecule structures. An alternative, used here, would be to use implicit water, that is, a continuum model, to mimic the bulk aqueous medium. All water molecules reported in the X‐ray analysis were included in the calculations. Klamt's COSMO^3^ technique has proven ideal for this purpose, and can simulate an aqueous environment by specifying that the liquid phase dielectric constant is 78.4. This has two advantages: it avoids all of the geometric problems associated with explicit solvent molecules, and it reduces the complexity of the self‐consistent field calculations.

The objective of this study was to investigate some of the issues that might compromise the simulation of mechanisms in protein chemistry; such mechanisms can be regarded as occurring on a landscape in which the two most important dimensions are energy and geometry. A prerequisite for any simulation involving such a landscape in that the simulated geometry should be of known accuracy but for *in vivo* protein work, there is no direct way of determining geometric error. A good approximation to the *in vivo* geometry is provided by X‐ray analysis; this forms the basis of an indirect measure of geometric accuracy. Instead of using a simple geometric change, the ratio of the change in energy between the geometry from X‐ray analysis and that predicted by a computational method can be used as an indirect, but more informative, measure of geometric accuracy. Chemistry is dominated by energy changes, and although geometry is, at least in principle, important, chemical processes are determined mainly by energy differences of reactants and products and by activation barriers. Because energy is so important, an error in the interatomic separation of two atoms that are bonded together should be given a much higher weight that an equivalent change in two atoms that are well separated. By using energy instead of geometry, these different types of geometric change are automatically taken into account. However, a disadvantage of using energy terms is that they are, of course, dimensionally different from geometry terms. This problem can be avoided by using ratios. The total change in energy in going from the X‐ray structure to the PM7 structure was split into two components: an error attributable to PM7, and the change in going from the X‐ray structure to the hypothetical ideal *in vivo* structure. The ratio of these two quantities then represents a comparison of the PM7 geometries to that of the X‐ray geometries regarding the similarity to the *in vivo* geometry. Before continuing, it should be reiterated that care must be exercised to avoid referring to the change in geometry in going from the X‐ray structure to the *in vivo* structure as an error. Even if X‐ray structures were completely accurate, because the crystal and solvent phase environments are different, there would necessarily be a change in the protein geometry.

In the survey of 19 proteins, the average total decrease in heat of formation per mole per atom in going from the X‐ray structure to the optimized PM7 structure was 0.98 kcal mol^−1^ atom^−1^ for gas‐phase and 0.78 kcal mol^−1^ atom^−1^ for solvent phase. This difference, −20%, can be attributed to the improvement in the model, in that the solvent‐phase model is more realistic than the gas‐phase model. The average total drop can then be partitioned into the contributions arising from the geometry change in going from the X‐ray structure to the HIM, and in going from the HIM to the PM7 fully optimized structure. By using high accuracy geometries as references, the average value for this latter change was found to be 0.12 kcal mol^−1^ atom^−1^ and for this work that value can be regarded as a constant. Given this constant, the former change, going from the X‐ray to HIM geometry, can then be worked out using Eqs. (3) and 4. For the gas phase, this amounts to 0.97 kcal mol^−1^ atom^−1^, and for the aqueous phase, 0.77 kcal mol^−1^ atom^−1^.

Almost all of the energy change resulting from optimizing an X‐ray structure can be assigned to changes in covalent bond lengths. When a constrained optimization was performed^15^ on the small protein Crambin, the constraint being a bias toward the original X‐ray structure, it was found that about 90% of the decrease in heat of formation was achieved when the root mean square change in atomic positions was ∼0.1 Å. Given that the total root mean square change in atomic positions was ∼0.7 Å, a six times larger change in geometry accounted for only 10% of the drop in heat of formation. This dramatically demonstrates the significance of the most important type of error found in X‐ray geometries: that even when the overall structure is very accurate, small geometric errors in the relative positions of covalently bonded atoms account for the majority of the energy drop in the heat of formation. As chemical processes are dominated by energetics, use of heats of formation seems an appropriate measure of accuracy.

When the proteins were examined, an error in PM7 was revealed. This was confirmed using the structure validation program MolProbity. After unconstrained geometry optimization some hydrogen–hydrogen separations became smaller than expected. This phenomenon did not occur when isolated pairs of amino acids were used, only when pairs of residues that would otherwise not interact were present in a protein, and then only when there was a nearby source of interaction, such as a hydrogen bond, that would tend to pull the residues together. That this was a fault in PM7 was confirmed when a B3LYP‐D3 simulation of the clash at 1.8 Å showed a potential of 1.7 kcal mol^−1^ where PM7 showed only 1.0 kcal mol^−1^ (Fig. 2).

This was an unexpected fault. To determine whether this fault was specific to PM7, the geometry of the test protein used in studying this error, 4MZC, was optimized using other semiempirical methods. The results showed that the error was present in most methods and that instead of the expected interatomic distance between two hydrogen atoms, one on Leu_104_ and one on Val_110_, of 2.24 Å, abnormally short distances were also predicted by PM6^16^ (1.78 Å), PM6‐DH2^17^ (1.78 Å), PM6‐DH+^18^ (1.64 Å), and PM3^19^ (1.77 Å). The only method tested that did not produce a clash was RM1,^20^ where the closest interhydrogen separation between the two residues in the optimized structure was 2.58 Å.

During the development of the semiempirical methods mentioned here, only equilibrium structures were used in the training set. No reference data representing anomalously close contacts were used, and as a result, this deficiency gave rise to an insensitivity to abnormally close interactions. This specific fault can be eliminated by including appropriate reference data in future parameterizations.

Having an error of this magnitude remain undetected for so long is unusual. In part, this can be explained by the very small energies involved. Examination of the two PES curves, Figure 2, shows that at the PM7 minimum (near 2.1 Å), the B3LYP‐D3 potential is only about 1.2 kcal mol^−1^. An energy this small would easily be overwhelmed when other noncovalent interactions, such as normal hydrogen bonds of about 5 kcal mol^−1^, are present. Only when there are no other interactions between the residues would this fault in PM7 become dominant and give rise to abnormally close contacts. This gives rise to a specific set of conditions that must exist in order for the error to appear:

- The fault would only appear in pairs of residues where the only interaction between them was hydrophobic. All other non‐covalent interactions would need to be absent.
- A potential small but attractive interaction had to be present on a nearby pair of residues, to pull the chains together. This is necessary to reduce the equilibrium H–H distance from ∼2.3 Å. In the case of 4MZC, this was provided by a hydrogen bond that existed in the optimized geometry but was absent in the X‐ray geometry.
- Other nearby strong hydrogen bonds, of the type that occur within alpha helices and beta sheets had to be absent. The presence of strong hydrogen bonds would make the environment too rigid to allow the motion needed for the error to become apparent.

Examination of several of the most severe clashes showed that in every case these conditions were met. These conditions would only occur in hairpin bends and disordered sections of proteins. Enzyme catalysis occurs at active sites and since these are usually highly conserved, the geometry of the site being stabilized by strong hydrogen bonds, it is unlikely that this error would manifest itself in investigations of enzyme mechanisms.

## CONCLUSION

For modeling chemical processes involving proteins, the use of energy changes provides a more useful measure of geometric distortions than simple atomic displacements. When the error in the modeling of *in vivo* protein geometries is represented as energy, the semiempirical method PM7 produces a distortion relative to a hypothetical ideal geometry of about 10% of the difference between the X‐ray structure and the ideal geometry. Although the difference between X‐ray and ideal geometries is much larger than the PM7 difference, about 90% of it is due to small displacements, on the order of 0.1 Å, in atom positions in the X‐ray structures. If this displacement is made to the X‐ray structures the remaining geometric difference between the X‐ray geometry and the ideal *in vivo* geometry is roughly the same as that between the predicted PM7 geometry and the ideal geometry.

A hitherto unreported error was found in some geometries predicted by PM7. This error, also found in other semiempirical methods, was traced to an under‐representation of the core‐core repulsion. It is unlikely that the error would have any significant quantitative effect on the modeling of processes that occur in proteins, and it should be straightforward to remove in future semiempirical methods.

## DISCLAIMER

This work is solely the responsibility of the authors and does not necessarily represent the official views of the National Institutes of Health.
